# Formal methods for safety-critical machine learning: a systematic literature review

**DOI:** 10.3389/frai.2026.1749956

**Published:** 2026-02-18

**Authors:** Alexandra Newcomb, Omar Ochoa

**Affiliations:** Department of Electrical Engineering and Computer Science, Embry-Riddle Aeronautical University, Daytona Beach, FL, United States

**Keywords:** formal methods, machine learning, safe autonomy, safety-critical systems, software verification

## Abstract

**Introduction:**

The integration of Machine Learning (ML) systems into safety-critical domains heightens the need for rigorous safety guarantees. Traditional testing-based verification techniques are insufficient for fully capturing the complex, data-driven, and non-deterministic behaviors of modern ML models. Therefore, applying formal methods—which provide rigorous mathematical guarantees of a system’s adherence to specified properties—to ML systems has been of particular interest in recent years.

**Methods:**

This work presents a comprehensive Systematic Literature Review of peer-reviewed research from 2020 to mid-2025 on the use of formal methods to enhance ML safety, specifically for safety-critical applications. Articles selected present empirical research applying formal methods to modern machine learning approaches. Application domains as well as gaps, limitations, and challenges in this research area are compiled and presented.

**Results:**

Following a structured protocol, 46 studies were identified across four major digital libraries and classified into eight categories: Reachability and Over-Approximation Techniques, SMT-based Verification and Abstraction/Refinement, MILP/ILP Approaches, Model Checking Approaches, Runtime Verification Approaches, Shielding Techniques, Control Barrier Function Methods, and Risk Verification Methods. The review synthesizes methodological advances, application areas, and comparative strengths over traditional verification, while also presenting bibliometric trends in the literature.

**Discussion:**

Analysis reveals persistent challenges and gaps, including scalability to large and complex models, integration with training processes, and limited real-world validation. Future research opportunities include developing integrated training-verification loops, scalable verification frameworks, hybrid formal methods, and novel techniques for emerging ML paradigms such as Large Language Models. This work serves both as a state-of-the-art reference and as a roadmap for advancing the safe deployment of ML systems.

## 1 Introduction

The increasing deployment of Machine Learning (ML) systems in critical sectors such as transportation, healthcare, and industrial automation introduces significant risks due to the inherent unpredictability and black-box nature of these complex models (Xiao et al., 2023; Bengio et al., 2025; Guendouzi et al., 2025; Adadi and Berrada, 2018). Failures in these systems can lead to severe consequences, including loss of human life, economic damage, and erosion of public trust. Furthermore, the rise of agentic AI warrants rigorous safety guarantees to ensure these systems work in the best interest of the public and are not susceptible to security breaches (Bengio et al., 2025). Therefore, safe ML has been identified as a critical area of future ML research. Notably, the first International Artificial Intelligence (AI) Safety Report, written as a collaboration by 96 AI experts following the first International AI Safety Summit in 2023, discusses the need for safety nets within ML systems (Bengio et al., 2025).

Traditional verification approaches, i.e., testing, struggle to provide comprehensive safety guarantees, especially given the data-driven and adaptive nature of ML algorithms (Samadi et al., 2024; Zhang et al., 2022; Meyer, 2023). Formal methods, encompassing rigorous mathematical techniques like Model Checking, Theorem Proving, and Runtime Verification, offer potential solutions to these challenges by enabling explicit reasoning about system correctness, safety properties, and robustness (Paul et al., 2023). This review defines formal verification as any rigorous mathematical method that reasons about a system’s behavior logically and provides theoretical guarantees against specified formal properties (Paul et al., 2023; Wang and Tepfenhart, 2020). Despite the promise of formal methods, the integration of formal methods into ML safety and quality assurance is challenging due to the non-deterministic and often unexplainable nature of ML models. Research within this area remains fragmented and underexplored, motivating the need for a systematic synthesis of existing research to clearly delineate the current state, capabilities, and limitations of formal methods for safe ML.

This work presents a Systematic Literature Review (SLR) of current research in the area of ML safety and quality assurance through the usage of formal methods. The SLR categorizes current work into eight categories: Reachability and Over-Approximation Techniques, SMT-based Verification and Abstraction/Refinement, MILP/ILP Approaches, Model Checking Approaches, Runtime Verification Approaches, Shielding Techniques, Control Barrier Function Methods, and Risk Verification Methods. Current literature belonging to each area is then detailed. Note that this SLR focuses specifically on ML safety through formal verification, and both explainability and LLM hallucination are therefore outside the scope of this review.

Section 2 details the research method of the SLR followed by both bibliometric and technical results presented in Sections 3 and 4, respectively. Section 5 presents the analysis and discussion of the extracted data. Section 6 summarizes the quality assessment results. Lastly, Section 7 presents related work, and Section 8 briefly discusses strengths and limitations of the methods.

## 2 Research method

This section details the protocol used to conduct the SLR. This protocol is based on the methodologies presented by Carrera-Rivera et al. (2022), Zhou et al. (2015), and Kitchenham and Charters (2007).

### 2.1 PICOC and synonyms

When framing the research questions and search terms, the Population, Intervention, Comparison, Outcome, and Context (PICOC) method was used Carrera-Rivera et al. (2022) and Kitchenham and Charters (2007). Table 1 presents the PICOC terms for this research.

**Table 1:** PICOC terms.

PICOC criteria	Term	Synonyms
Population	Machine learning	Artificial intelligence, neural network, reinforcement learning, large language model, transformer
Intervention	Formal methods	Formal verification, temporal logic, model checking, theorem proving, runtime verification, reactive synthesis, static analysis, automated reasoning
Comparison	Testing	Traditional verification
Outcome	Safety	Quality, safe
Context	Safety-critical	Critical system, life-critical, mission-critical, high-reliability

### 2.2 Research questions

This research addresses the following three Research Questions (RQs), formulated from the PICOC terms.

1. What formal methods are currently used to ensure the safety of ML systems and how do they improve upon traditional verification approaches?
2. In which domains or applications have these formal methods been successfully employed for safe ML?
3. What gaps, limitations, and challenges exist within current work combining formal methods and ML safety, and what future research directions can be identified from these gaps?

### 2.3 Digital library sources

Articles for the SLR were selected from IEEE Xplore, ACM Digital Library, Science Direct by Elsevier, and Springer. These digital libraries were chosen for their relevance to the RQs and their reputability for publishing peer reviewed articles.

### 2.4 Search strings

Using the PICOC criteria, the following search string was synthesized: (“Machine Learning” OR “Artificial Intelligence” OR “Neural Network” OR “Reinforcement Learning” OR “Large Language Model” OR “Transformer”) AND (“Formal Methods” OR “Formal Verification” OR “Temporal Logic” OR “Model Checking” OR “Theorem Proving” OR “Runtime Verification” OR “Reactive Synthesis” OR “Automated Reasoning”) AND (“Testing” OR “Traditional Verification”) AND (“Safety” OR “Quality” OR “Safe”) AND (“Safety-Critical” OR “Critical System” OR “Life-Critical” OR “Mission-Critical” OR “High-Reliability”).

An additional title search term was used for Springer to narrow down results further: “Formal” OR “Temporal Logic” OR “Verification” OR “Model Checking” OR “Runtime.” Because Science Direct only allows eight Boolean connectors per query, the search string was split into the following two search strings, for Science Direct only:

1. (“Machine Learning” OR “Artificial Intelligence” OR “Neural Network”) AND (“Formal Methods” OR “Formal Verification”) AND (“Testing”) AND (“Safety”) AND (“Safety-Critical”)
2. (“Reinforcement Learning” OR “Large Language Model” OR “Transformer”) AND (“Model Checking” OR “Runtime Verification”) AND (“Testing”) AND (“Quality” OR “Safe”) AND (“Safety-Critical”).

### 2.5 Inclusion and exclusion criteria

The inclusion and exclusion criteria are presented in Table 2. Criteria are adapted from Carrera-Rivera et al. (2022) with several additions. Due to the significant development in ML in the last five years, articles were chosen to be no earlier than 2020. Despite their lower quality as opposed to conference and journal articles, workshop articles were included, as these articles frequently present novel ideas for future exploration.

**Table 2:** Inclusion and exclusion criteria.

Criteria	Inclusion	Exclusion
Time Period	2020 to June 2025 (5.5 years)	Articles published before 2015 and after June 2025.
Language	Articles written in English.	All articles not written in English.
Type of literature/source	Conference, workshop, and journal articles presenting original empirical research.	All other articles, including grey literature. Articles that do not present empirical research.
Relevance to Research Questions	Relevant articles to at least one research question.	Articles irrelevant to all research questions

In addition to the criteria presented in Table 2, studies that do not directly apply formal methods to ML systems were excluded. This exclusion applies to studies that formally verify controllers or code synthesized by an ML model, or to studies focusing on autonomous systems broadly. Articles that do not mention “formal verification” or “formal methods” explicitly were removed. Articles related to cybersecurity were also excluded, as this SLR focuses on safe ML as opposed to secure ML.

### 2.6 Quality assessment checklist

After the initial articles were selected using the inclusion and exclusion criteria on the full list, the quality assessment checklist, shown in Table 3, was used primarily to characterize the quality of the articles and help determine future research directions. Articles were not excluded based on the quality assessment criteria, as this work is primarily interested in the ideas and research directions within safe ML through formal methods. Additionally, due to the limited nature of work in this area, it was counterproductive to further exclude articles outside of the inclusion and exclusion criteria. The questions are adapted from Zhou et al. and utilize the Reporting, Rigor, Credibility, and Relevance criteria (Zhou et al., 2015). For each article, each question received a score of either 0 – “No,” 0.5 – “Partially,” or 1 – “Yes.”

**Table 3:** Quality assessment checklist.

ID	Quality assessment question
	*Reporting*
1	Do the authors clearly state the aims (goals, purpose, problems, motivations, objectives, questions) of the research?
2	Does the study clearly answer the research question(s) or clearly present the results?
	*Rigor*
3	Are the methods used in the research clearly stated and fully defined?
4	Was the data collection method sufficiently rigorous and clearly described?
5	Does the research design address the aims of the research?
6	*Credibility*
7	Does the article discuss limitations, challenges, or threats to validity?
8	Is the research replicable?
9	Are the findings credible (free from bias, reliable, trustworthy)?
	*Relevance*
10	Is the study relevant or of value to the research community?

### 2.7 Data extraction form

After obtaining the final set of research articles, data was systematically extracted for analysis using the data extraction form presented in Supplementary Table 1. The data extraction form was generated using guidelines from Kitchenham and Charters (2007).

Note that fields related to experimental design and results were not directly used to answer the RQs. Extracting information about each experiment was used to validate the significance of the authors’ findings and therefore the relevance of the proposed method. As a result, these fields primarily assisted with the quality assessment of each study. Experimental data was also used to help determine the application area or domain when answering RQ2.

### 2.8 Data analysis and synthesis

After extracting data using the data extraction form, the data was analyzed and synthesized through two methods. First, a bibliometric analysis was performed on the data to determine bibliometric information such as which conferences or journals produce the most amount of research in this area, the top three countries where research in this area is being conducted, and the citation counts for the different articles.

Next, the technical content from the articles was analyzed from the extracted data to answer the RQs. This was accomplished by comparing the responses to each data item across articles to determine trends, application areas, challenges, and future research directions. The findings are then synthesized to answer the RQs in Sections 4 and 5. Data is presented both quantitatively and qualitatively. Separately, an analysis of the quality of the articles is presented after executing the quality assessment checklist on each article.

## 3 Bibliometric results

**Figure 1:**
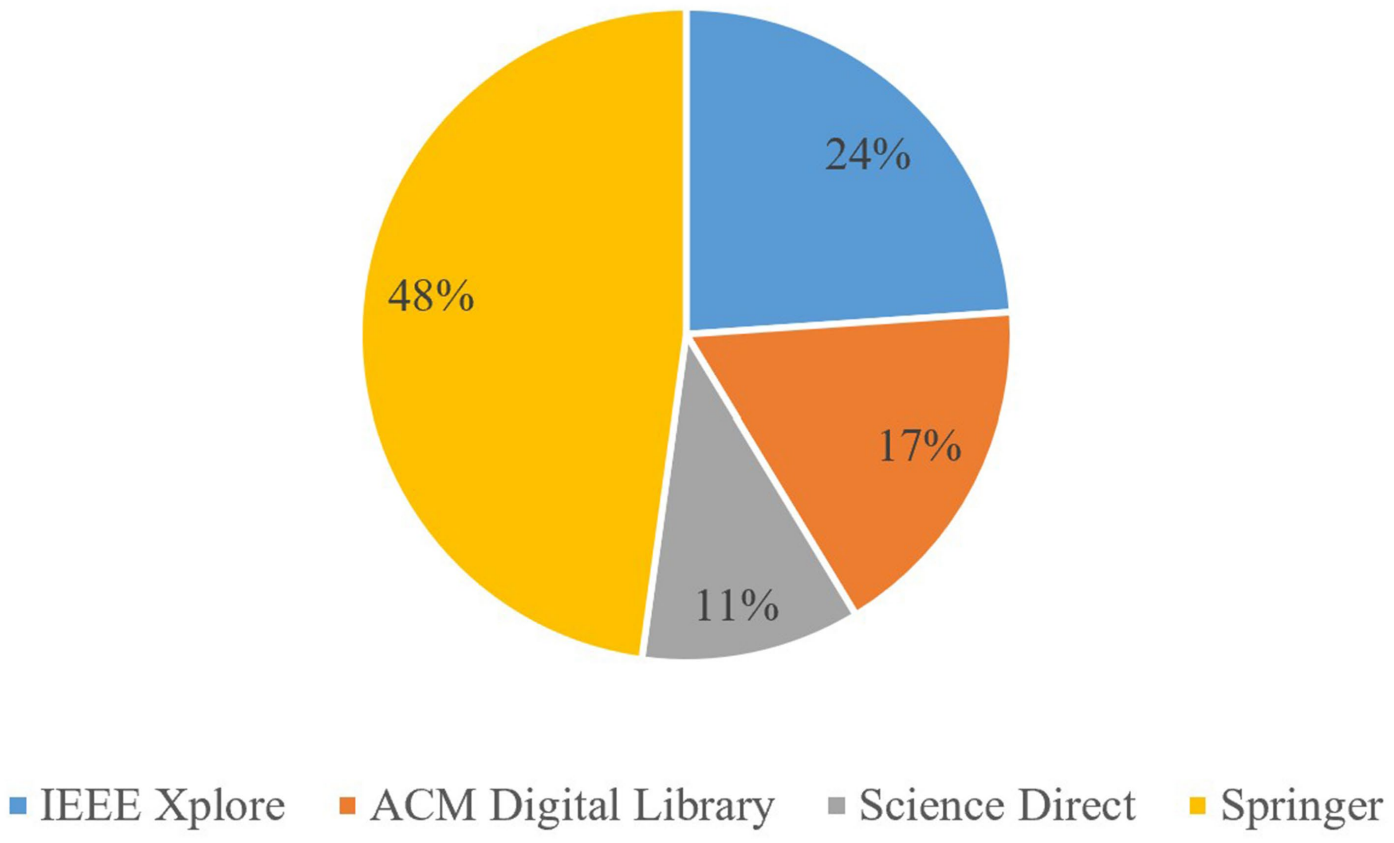
Distribution of selected articles from digital library sources.

When querying the respective databases with the search string, the number of papers listed were 215 for IEEE Xplore, 300 for ACM Digital Library, 266 for Science Direct, and 135 for Springer. This resulted in a total of 916 papers. The addition of the title search term for Springer narrowed results down considerably from 705 initial articles from Springer alone. The advanced search feature was used in each digital library to filter by year and for research articles. After the initial selection of papers, abstracts and titles were read to determine relevance to the RQs. As a result, the number of articles ultimately selected for the SLR were 11 from IEEE Xplore, 8 from ACM Digital Library, 5 from Science Direct, and 22 from Springer. The distribution of papers by digital library source is shown in Figure 1.

**Figure 2:**
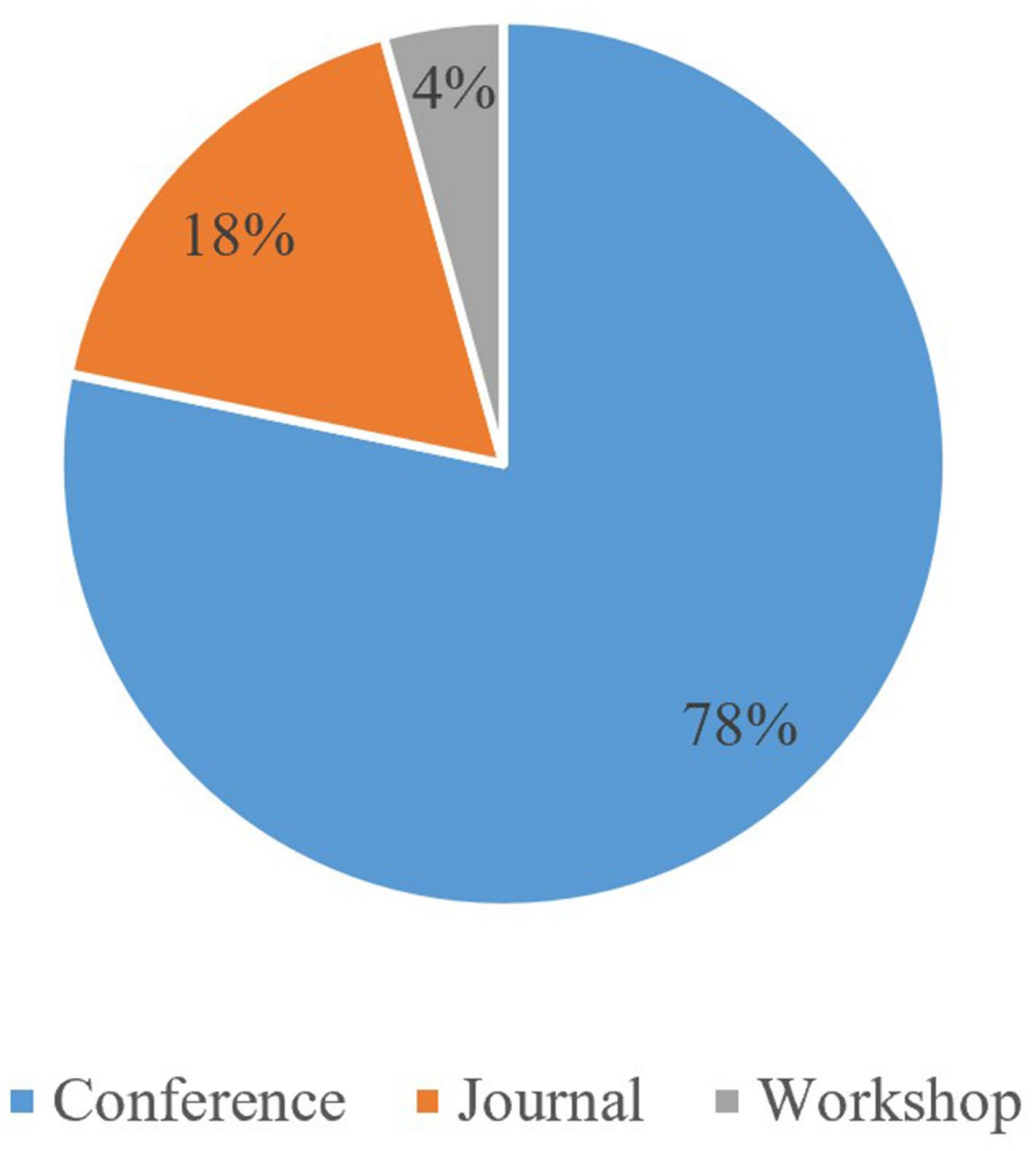
Distribution of articles selected as conference, journal, or workshop articles.

The distribution of selected articles that were published as conference papers, journal articles, or workshop papers is shown in Figure 2. Most reviewed articles were conference papers belonging to 26 distinct international conferences. The largest number of conference papers came from Bridging the Gap between AI and Reality, published on Springer. Journal articles similarly came from a variety of journals, with no single journal producing multiple articles used in this SLR. Only two workshop articles were selected for review, and both were published as part of the International Workshop on Verification and Monitoring at Runtime Execution (VORTEX) proceedings, published on ACM.

**Figure 3:**
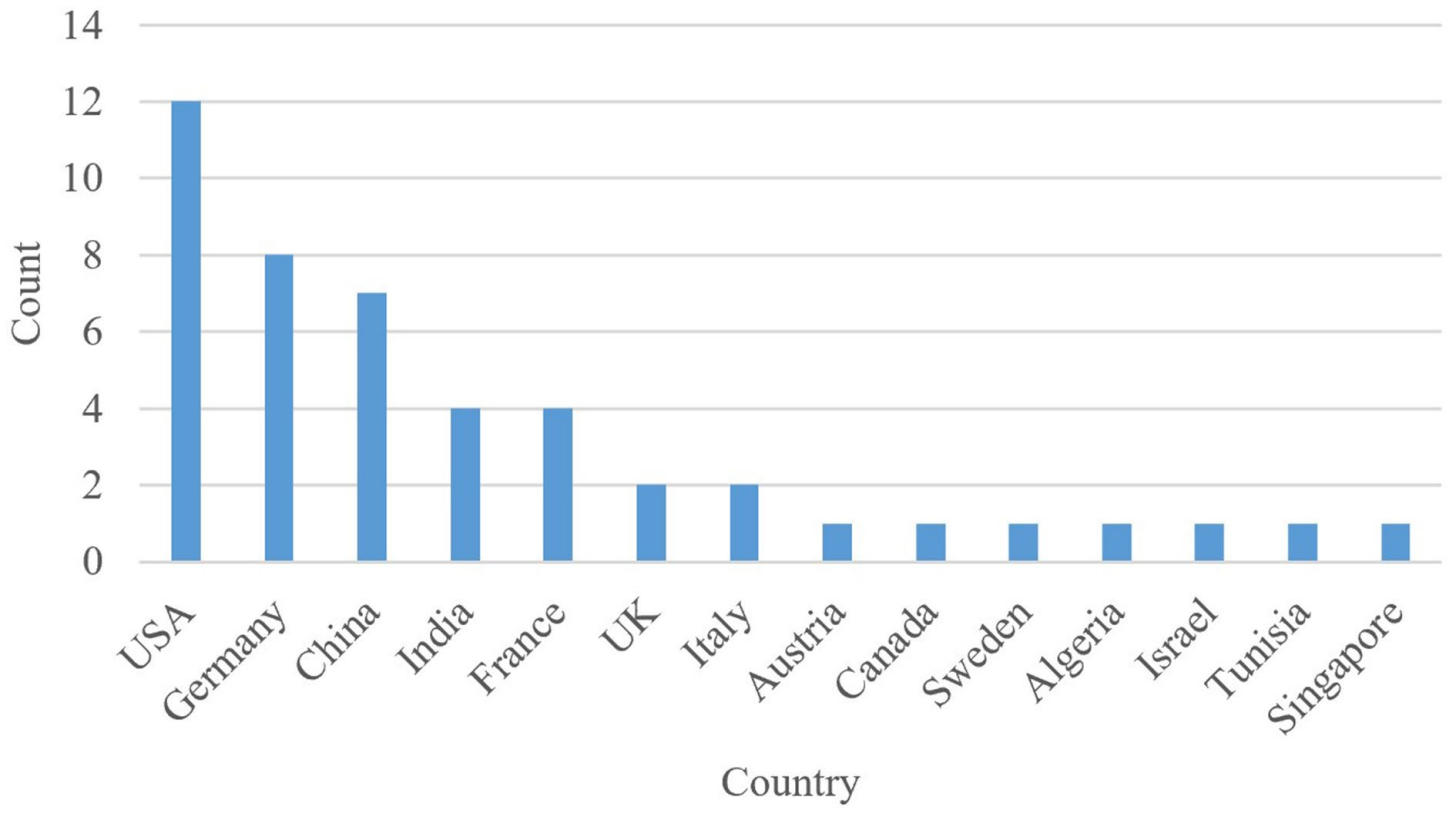
Distribution of selected articles by country.

**Figure 4:**
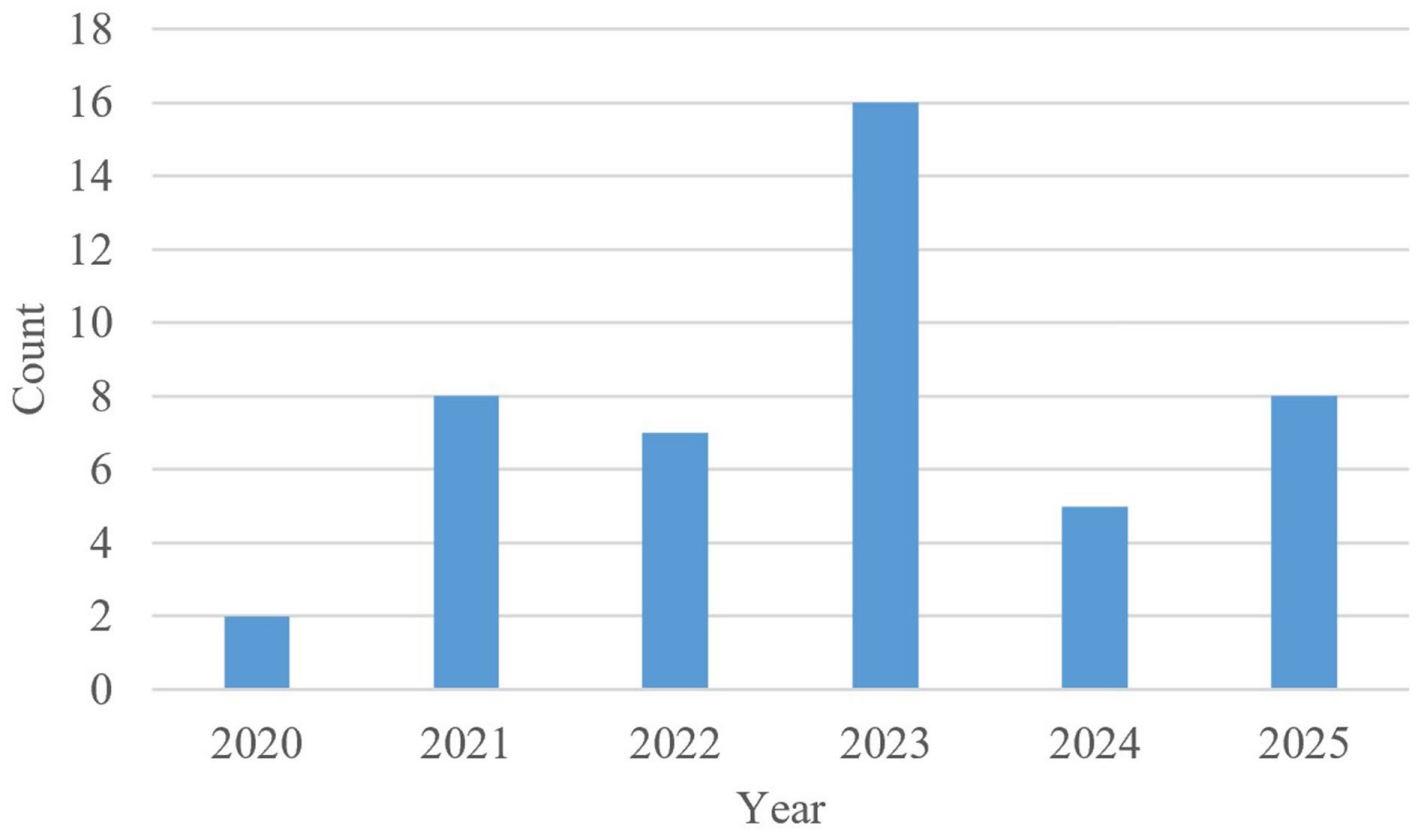
Distribution of selected articles by year.

**Figure 5:**
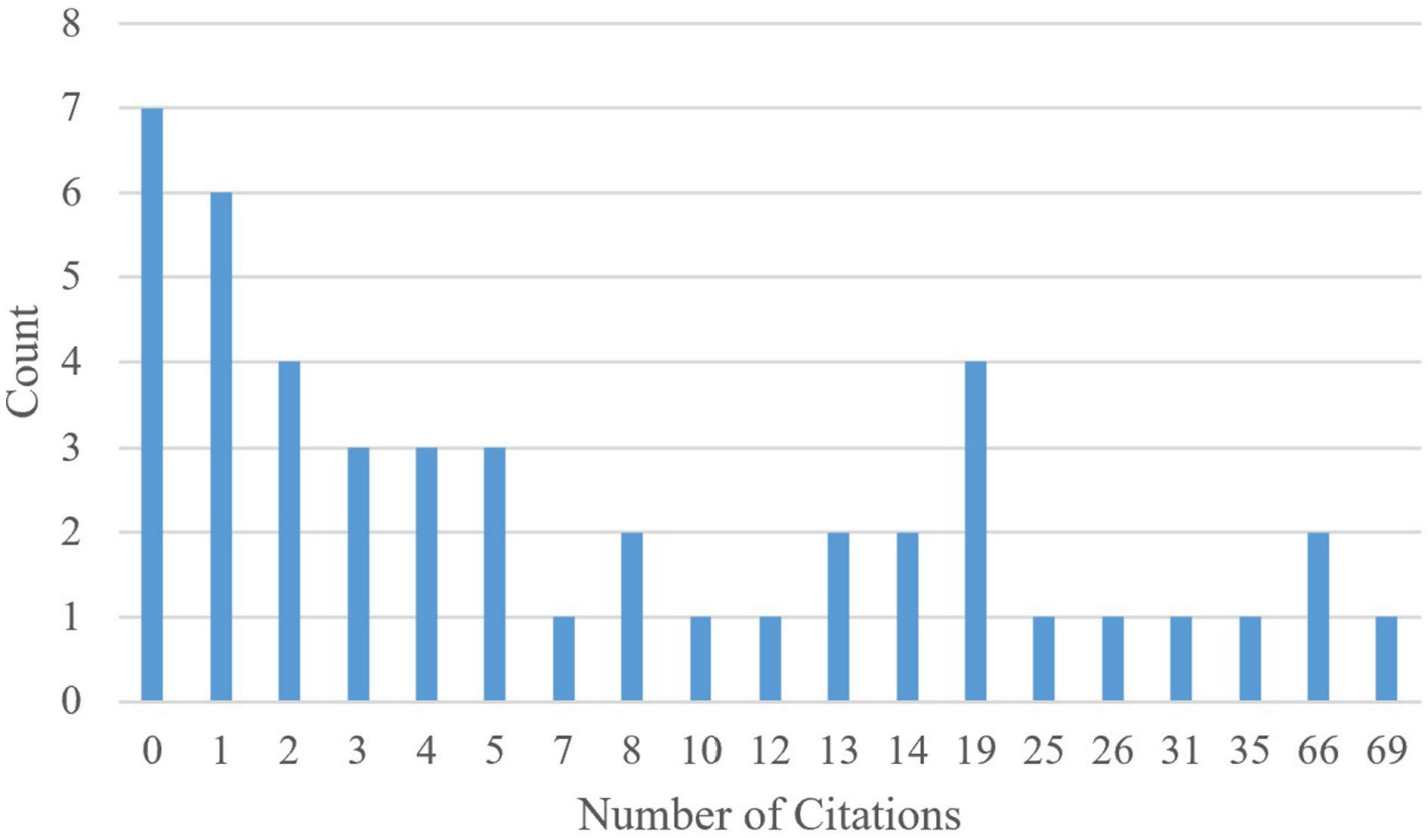
Distribution of citation counts for the selected articles.

The countries of origin of the selected articles are shown in Figure 3. The United States of America and United Kingdom are shortened to USA and UK, respectively. The top three countries from which the largest number of articles originated from are the USA, Germany, and China. Furthermore, Figure 4 displays the distribution of articles by year. Interestingly, the number of articles on the subject of safe ML through formal methods has remained steady over the last five years, with the largest number of articles published in 2023 and the lowest number published in 2020. It is noteworthy that a significant number of the articles were published in 2025, as only the first six months of 2025 were included in the search. Lastly, the distribution of citation counts for the selected articles is shown in Figure 5. Because many of the articles are new, i.e., published within the last two years, there are many articles with zero citations.

## 4 Technical results

Supplementary Table 2 summarizes the articles discussed throughout the rest of this work. Articles are referred to by their Identifications (IDs), where “S” stands for “Study.” After data extraction, findings from the studies were grouped into the following eight categories, which are used to answer RQ1 in the rest of this section. While some articles fit under multiple categories, each article was classified under the most applicable category. The eight categories of formal methods identified to ensure the safety of ML systems are:

1. Reachability and Over-Approximation Techniques
2. SMT-based Verification and Abstraction/Refinement
3. Mixed Integer Linear Programming (MILP)/Integer Linear Programming (ILP) Approaches
4. Model Checking Approaches
5. Runtime Verification Approaches
6. Shielding Techniques
7. Control Barrier Function Methods
8. Risk Verification Methods

### 4.1 Reachability and over-approximation techniques

Reachability analysis for neural networks computes the set of all outputs the network can produce when its inputs—and, if modeled, its parameters (weights and biases)—vary within specified bounds. This technique propagates sets (e.g., represented by intervals or star sets) through layers or time steps; some solutions compute a sound over-approximation that provably contains every true output, while other architectures admit exact set propagation (Meyer, 2023; Choi et al., 2025).

S22 extends the star-set reachability framework to RNNs to aid in formally verifying the robustness of RNNs (Choi et al., 2025). The authors develop both exact and over-approximate reachability algorithms that incrementally unroll recurrent layers, encode dependencies between current and past hidden states via Minkowski sum of star sets, and offer soundness and completeness guarantees. Unrolling refers to the technique of expanding the recurrent network across a chosen number of time steps by creating a separate copy of the recurrent layer for each time step. This transformation converts the RNN’s cyclic, time-dependent structure into a deep, acyclic Feed-Forward Neural Network (FFNN) at the cost of producing a much larger network (Choi et al., 2025).

S23 proposes a mixed-monotonicity-based reachability method for computing interval over-approximations of a NN’s output when both its inputs and its parameters lie within known bounds (Meyer, 2023). In this approach, the network’s partial networks are treated as static functions whose derivatives are known to lie within a certain bound. For each output neuron, a worst-case input corner is chosen based on whether each slope is positive or negative. The input corners are then used to compute tight upper and lower output bounds. This approach handles uncertainties by treating both input values and all weights as uncertain within given intervals and uses interval arithmetic on the network’s Jacobian to propagate these uncertainties layer by layer. Rather than completing this step once, the method is applied to every contiguous block of layers (i.e., all partial networks). These interval bounds are then intersected to result in a tighter overall output range. This approach is applicable to any Lipschitz-continuous activation function (Meyer, 2023).

On the other hand, S28 presents a high-parallelization framework for formally verifying feed-forward DNNs under bounded input uncertainty by computing interval over-approximations of layer outputs (Hafaiedh et al., 2025). Interval over-approximation is a static analysis technique that computes, for each neuron in the network, a conservative lower-upper bound on its activation by propagating input uncertainty through the network. The framework checks if, for all inputs satisfying some precondition, the DNN’s outputs satisfy a specified post-condition. The method runs several incomplete verifiers in parallel, and for each neuron takes the intersection of their interval bounds to refine the layer’s over-approximation before feeding it forward (Hafaiedh et al., 2025).

S12, S29, S31, and S34 apply reachability and over-approximation techniques to NNs for image-based applications (Parameshwaran and Wang, 2025; Zhong et al., 2023; Tang et al., 2023; Ashok et al., 2020). S12 aims to improve the scalability and efficiency of the verification problem for image-based NNs by performing formal verification, specifically linear bound propagation using 𝛼 − 𝛽 – 𝐶𝑅𝑂𝑊𝑁, on structured input spaces within the latent space. This technique thereby reduces input dimensionality and computational complexity. Specifically, the authors propose the Scalable and Interpretable Verification of Image-Based Neural Network Controllers (SEVIN). The method learns a structured latent representation of the controller’s input space, thereby decreasing the computational complexity of the verification task and making formal verification more scalable (Parameshwaran and Wang, 2025).

Similarly, S29 introduces the Abstract Refinement Enhancer for Neural network verifAcation (ARENA), which constructs a linear constraint encoding of a NN’s behavior using abstract interpretation bounds, then iteratively refines that encoding. ARENA targets potential violations of robustness and proves whether the regions are spurious (Zhong et al., 2023). S31 introduces the tool Fast Grouping for Multi-neuron Relaxation (FaGMR), a verifier that encodes the network and an input-perturbation region into linear constraints. The authors also address verifying robustness properties. Nonlinear activations are over-approximated by convex constraints, then a Linear Programming (LP) solver checks whether the predicted class can change within the perturbation region (Tang et al., 2023). S34 proposes DeepAbstract, an abstraction-based method that clusters neurons within each hidden layer based on empirical behavior (Ashok et al., 2020). Each neuron is represented by its vector of activation values over a chosen input set, then k-means groups neurons with similar activation vectors. Neurons in each cluster are merged, yielding a smaller abstract network. Verification is then run on the abstract network.

On the other hand, S30 and S43 focus specifically on Semantic Segmentation NNs (Pal et al., 2023; Tran et al., 2021). S30 performs set-based reachability analysis for neural networks, specifically star-set reachability using the “approx-star” method (Pal et al., 2023). The work presents a standardized benchmark intended to support fair and repeatable comparisons of verification approaches for semantic segmentation. S43 performs reachability analysis on Semantic Segmentation NNs, using ImageStar set representations and LP optimization to compute bounds needed for over-approximation (Tran et al., 2021). The authors introduce a relaxed reachability variant for Rectified Linear Unit (ReLU) layers and pixel-classification reasoning, which is controlled by a relaxation factor that reduces how many LP problems are solved.

S37 focus specifically on reachability analysis and abstract interpretation for CNNs (Kirov et al., 2023). The CNN’s safety-relevant requirements are formalized as mathematical properties over bounded input perturbation sets and bounded trajectory modification sets. For each property, the verifier over-approximates the CNN’s reachable output set over the constrained input set and checks whether the output set violates the output bound. When over-approximation yields “unknown,” randomized simulation searches for an explicit violating example.

S41 instead explores safety assurance when quantizing DNNs for use on resource constrained devices (Zhang et al., 2023). The authors use both Differential Reachability Analysis (DRA) and MILP. Given a DNN, its corresponding fully quantized QNN, an input region, and an allowed error bound, the method verifies whether the maximum output deviation between the DNN and QNN is always less than the bound for all inputs in the region. DRA attempts to prove the bound quickly; if it cannot, the MILP encoding is used to decide the property exactly.

Lastly, S42 applies star-based reachability analysis to DNNs used for time-series regression tasks (Pal et al., 2023). The framework encodes bounded sensor noise as a set of possible inputs and then propagates that set through the network layer-by-layer to obtain an output reachable set. The reachable output bounds are then compared to permissible bounds to determine robustness at each time step and across a sequence.

### 4.2 SMT-based verification and abstraction/refinement

SMT-based verification for NNs encodes both the neural network’s computation and the safety or robustness property as logical formulas in rich theories (e.g., linear real arithmetic or bit-vectors) and then utilizes optimized SMT solvers to check for violations or to prove the absence of counterexamples (Das et al., 2025; Bachiri et al., 2025). Abstraction/Refinement (AR) verifies a DNN by first constructing a smaller over-approximating model whose correctness implies correctness of the original. If the abstract query is UNSAT, the original is UNSAT; if it is SAT, the produced counterexample is checked on the original network and refinement is applied only for spurious counterexamples or otherwise inconclusive abstract results. By iteratively refining the abstraction, AR trades precision for solver performance and can be substantially faster than verifying the full network directly (Elboher et al., 2024).

S3, S4, S27, S32, and S35 specifically apply SMT-based verification to DNNs (Nuhu et al., 2022; Samadi et al., 2024; Elboher et al., 2024; Zhao et al., 2022; Paterson et al., 2021). After determining candidate unsafe input subregions (i.e., candidate unsafe sub-requirements), S3 utilizes the Marabou framework to validate the unsafe input space on a DNN (Nuhu et al., 2022). Marabou, an SMT-based framework for verifying DNNs, validates whether the input subregion violates the safety property. The Negative Selection Algorithm, a meta-heuristic algorithm, is used to search for candidate unsafe input subregions. Next, the subregions are fed into Marabou to formally verify that the corresponding safety property is violated, thereby validating the unsafe subregion and unsafe sub-requirement.

Similarly, S4 uses Marabou to systematically insert perturbations into the DNN under test and determine which safety properties are violated as a result (Samadi et al., 2024). The authors propose utilizing Marabou to analyze the resilience of a DNN to input perturbations. Specifically, the study aims to determine the threshold weights that result in property violations and focuses on Single Event Upsets (SEUs) and Multi-Bit Upsets (MBUs). An SEU is a single bit flip within the memory’s stored data, which impacts the weights of the DNN parameters. SEUs are performed by randomly selecting a weight within the DNN and flipping a bit in its binary representation. An MBU occurs when multiple bits are flipped in the system memory or processor of the DNN due to multiple SEUs in the network parameters. The proposed framework simulates both SEUs and MBUs within DNN network parameters. The authors then analyze the resulting effect on DNN performance and adherence to properties. The output of the DNN is compared with the anticipated output from the training data.

**Figure 6:**
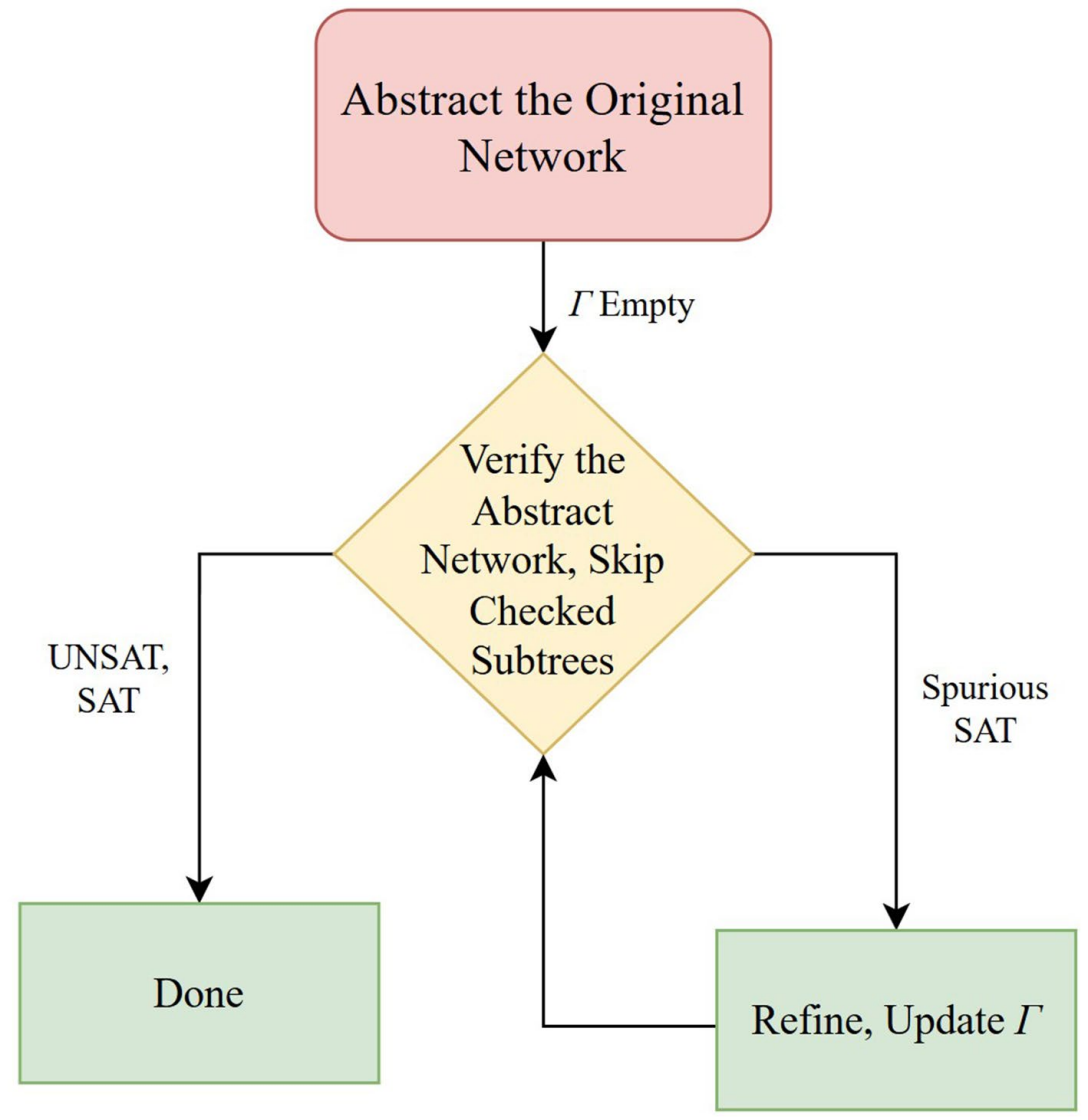
Verification loop as described in S27 (Elboher et al., 2024).

S27 focuses on AR for SMT-solving by introducing residual reasoning, a scheme that captures and reuses information (e.g., already-proven safe regions of the search tree) across successive AR iterations, thereby pruning redundant work and accelerating verification of DNNs (Elboher et al., 2024). The authors contribute a formal, sound, and complete residual-reasoning framework for DNN verification as well as a detailed design for extending the Marabou verifier to support residual reasoning. Figure 6 displays the verification loop described by S27, where Γ is a context formula encoding scenarios already shown safe.

S32 applies Counterexample-Guided Abstraction Refinement (CEGAR) to DNN verification to determine whether a DNN satisfies specified input/output safety properties formulated as verification queries (Zhao et al., 2022). The framework involves building an over-approximated abstract network of the DNN and iterating AR using counterexamples. Backend formal verification is accomplished using Marabou. S35 introduces DeepCert, which performs constraint-based formal verification of DNN robustness using Marabou (Paterson et al., 2021). DeepCert encodes a perturbation to an image as constraints linking original pixels, perturbed inputs, and a perturbation bound, then determines whether a misclassification is possible within that bound.

On the other hand, S19 focuses on verifying the robustness of SDNNs, in safety-critical applications, from adversarial attacks using formal verification (Das et al., 2025). The authors specifically explore modeling and verification using SMT constraints. The framework receives two inputs: the SDNN and a property for verification. The formal verification framework then rigorously analyzes the SDNN using SMT-solving and either declares property adherence or presents a counterexample. The authors also present an interval bound derivation to improve the performance of the SMT solution further.

S20 presents an SMT-based verification of QNNs by approximating QNN values with rational-number versions (Bachiri et al., 2025). The authors combine rational approximations using set theory with SMT-based verification over rational arithmetic programs. The authors’ approach consists of four steps:

1. Convert natural language descriptions of how an autonomous vehicle controller should behave into formal SMT predicates. This is done by first converting textual requirements into abstract scenarios that describe the intended autonomous driving behaviors. Then, these abstract scenarios are translated into logical scenarios from which formal decision properties can be specified.
2. Approximate the fixed-point QNN with a rational-number version by replacing every weight, bias, and operation with its exact rational-number counterpart. This supports SMT solving, as fixed-point mathematics is computationally infeasible for SMT solving at scale.
3. Verify the safety property over the rational network using SMT-LIB. If the solver returns UNSAT, no counterexample exists. If the solver returns SAT, there is an input violating the property, and the check stops.
4. Generalize the results back to the original QNN by accounting for quantization error that the fixed-point QNN experiences during arithmetic operations (Bachiri et al., 2025).

On a separate note, S33 models a NN controller as a transition predicate and utilizes an inductive invariant method for proving time-unbounded safety (Zhou and Tripakis, 2024). The framework decomposes the verification step so that the NN-related implication is handled using an NN verification engine and the environment-related implication is handled using an SMT solver.

S39 formally verifies an RL-trained controller for vertical collision avoidance (Genin et al., 2021). The authors compute conservative safe command ranges for states in a critical region, discretize the state space into cubes and over-approximate the safe bounds per cube, then use an SMT solver to prove whether the NN’s command acceleration is always within safe bounds for each critical cube. The result is a classification of cubes as verified safe versus potentially unsafe.

Focusing on a different area of ML research, S45 applies SMT solving to Spiking NNs, which are NNs that process information via discrete “spikes” over time (similar to human brains) (Banerjee et al., 2023). The framework translates the step-by-step execution of a Spiking NN into SMT constraints, where Boolean variables represent whether each neuron spikes at each timestep, and real variables represent each neuron’s instant potential and stored potential. Properties are then verified by checking satisfiability of a combined constraint system representing the Spiking NN behavior, input constraints, and violation of the desired output condition. If satisfiable, the solver provides a counterexample input spike train. If unsatisfiable, the property holds for all allowed inputs.

Lastly, S46 focuses specifically on SMT solving for Long Short-Term Memory (LSTM) networks, which are a type of RNN designed for sequential and time-series data (Moradkhani et al., 2023). The authors train an LSTM model, extract learned parameters, and encode the LSTM’s step-to-step update equations as an SMT constraint system. They then use an SMT solver with Bounded Model Checking capabilities to determine whether the LSTM can violate the intended safety specification.

### 4.3 MILP/ILP approaches

MILP/ILP verification for NNs encodes a NN and a safety specification over a bounded input set into a set of linear and integer constraints/inequalities. Integer (binary) variables capture discrete choices introduced by activations and quantization; the solver asks a feasibility question: “Is there an input in this set that violates the property?” If the solver finds one, that assignment is a concrete counterexample; if none exists, the property is certified for that region. MILP mixes continuous and integer variables, whereas ILP uses only integers (Zhang et al., 2022; Mistry et al., 2022).

S5 presents an approach in which fixed-point primitives are encoded as operations in MILP to allow the authors to apply MILP solving to QNNs (Mistry et al., 2022). Otherwise, MILP cannot be used directly on QNNs. The verification problem is converted from a validity problem to a satisfiability problem, which allows the MILP solver to determine satisfaction (with a produced counterexample) or unsatisfaction (indicating a successful verification or valid input formula). Rather than being used for optimization, the MILP solver is used to check the feasibility of the equations. Similarly, S14 presents an encoding method for QNNs that reduces the verification problem to ILP (Zhang et al., 2022). The authors introduce piecewise constant functions for the encoding of QNN activation functions, which are then further encoded as integer linear constraints using additional Boolean variables. Both S5 and S14 use Gurobi as the backend MILP/ILP solver.

Separately, S36 applies MILP to ReLU NNs to verify monotonicity properties for a safety-critical avionics system (Vidot et al., 2022). A model is monotone if an input change in a specified direction results in an output moving in only one direction. The authors encode the ReLU NN and the monotonicity requirement into MILP constraints. They then check monotonicity over many paired input sub-spaces. By running MILP feasibility checks on each sub-space, they classify regions where monotonicity holds, fails everywhere, or partially fails.

Lastly, S6 combines polynomial inclusion computation with barrier certificate generation to formally verify synthesized DNNs from RL using linear programming solvers (Zhao et al., 2023). The authors abstract a DNN controller as a polynomial using Bernstein polynomials to facilitate verification by polynomial inclusion. Safety adherence is determined by the existence of barrier certificates under the abstract controller, and Sum-of-Squares optimization is used to search for these barrier certificates. If a barrier certificate is found, adherence to the corresponding safety property is determined for all executions of the original DNN controller.

### 4.4 Model checking approaches

Within formal methods broadly, Model Checking provides an automated framework to verify that system designs meet desired specifications. An abstract model *M* of the system under verification is constructed using finite state automata. Programs are modeled as transition systems, represented using nodes, variables, and transitions. A set of correctness and safety formulas *F* is defined using a logic formalism. The formulas describe the required correctness properties of the system. Next, the state space of *M* is systematically explored to check if *F* is consistently satisfied. If a property is violated, the model checker provides a counterexample demonstrating where the violation occurs (Paul et al., 2023; Jhala and Majumdar, 2009).

S2 applies Symbolic Model Checking to NNs by formally modeling a trained NN and verifying the model to estimate robustness to noise (Naseer et al., 2020). The study introduces the Formal Analysis of Neural Network (FANNet), which is composed of three main procedures. The first procedure is behavior extraction, in which the formal model of the NN is built using the weights and activations of the network on known test samples. The temporal properties are translated into the logical language of the model checker. The second procedure is noise tolerance analysis, in which the specific noise tolerance for the NN is algorithmically determined. The last procedure is the adversarial noise vector extraction, in which a unique array of noise patterns that the NN is sensitive to is built (Naseer et al., 2020). S9 applies Bounded Model Checking to a simplified version of a DNN modeling adaptive cruise control behavior (Nenchev, 2025). However, the model checked may not describe all behaviors of the actual system, therefore introducing potential gaps in the evaluation. The proposed approach should therefore be used alongside other verification techniques.

Both S1 and S44 apply Model Checking to RL-based systems (Tao et al., 2025; Adelt et al., 2023). S1 proposes a framework for formally specifying requirements, constructing an abstract model, and model checking an RL-based system (Tao et al., 2025). The study provides specification templates for formally specifying requirements of RL-based systems. A model construction process for generating the abstract model is then presented. Finally, the authors present Reinforcement Learning Verification-as-a-Service (ReLVaaS), a framework allowing users to specify formal requirements, construct the abstract models, and perform model checking using PRISM and Storm. On the other hand, S44 combines shielding with Statistical Model Checking-based learning for an RL agent (Adelt et al., 2023). The RL agent is constrained by a verified shield, and the authors prove, in Differential Dynamic Logic (dL) and KeYmaera X, that actions satisfying the agent’s contracts preserve safety and resilience. The framework then enforces, at runtime, that the agent can only choose those safe actions. Statistical Model Checking is used both during training and for statistical evaluation to compute confidence intervals of property satisfaction.

S26 introduces Deep Statistical Model Checking (DSMC), a framework that treats a trained neural network as an oracle to resolve nondeterminism in a Markov Decision Process (MDP), yielding a Markov chain whose behavior can be assessed via Statistical Model Checking (Gros et al., 2022). DSMC treats a trained neural network as a black-box oracle that resolves nondeterministic choices in a formally specified MDP. Whenever the MDP reaches a decision point, it queries the NN, using the current state, for the next action. The result is a fully probabilistic Markov Chain that can then be used for analysis.

On a separate note, S25 presents a unified Model Checking framework for ReLU RNNs that leverages a polyhedron abstraction domain and bidirectional propagation to symbolically verify both qualitative and quantitative temporal and robustness properties (Liang et al., 2024). The contribution is a systematic verification framework that combines polyhedron forward propagation, dimension preserving abstraction, and Monte Carlo sampling. Polyhedron forward propagation is used to track sets of possible RNN states. Dimension-preserving abstraction is used to curb combinatorial explosion of polyhedral vertices. Lastly, Monte Carlo sampling and backward propagation are used for quantitative estimation of satisfaction probabilities and to refine approximations.

Model Checking of MARL frameworks is explored by S15, which applies Model Checking to an abstract representation of a Markov game and joint policies (El Mqirmi et al., 2021). The study presents Assured Multi-Agent Reinforcement Learning (AMARL), a formal verification framework for MARL systems. AMARL formally guarantees the safety of agents acting in an unknown environment. The authors introduce the Abstract Markov Game (AMG) and present a procedure for automatically generating AMGs to conduct verification over.

S21 applies Model Checking to a framework combining Federated Learning, Genetic Algorithms (GA), and Meta-learning into a multi-layer Industrial Cyber Physical Systems (ICPS) framework named FedGA-Meta (Guendouzi et al., 2025). The authors first model the ICPS. Key elements of the ICPS, such as sensors, actuators, edge/fog/cloud servers, the network links, the data flows, and the Federated Learning process itself, are formalized as a Labeled Transition System (LTS). The behavioral models are then represented as timed automata in UPPAAL, and each property is posed as a query to the UPPAAL model checker. UPPAAL then exhaustively explores every possible execution within specified timing bounds to verify that each property holds, producing a counterexample otherwise. This provides a machine-checked guarantee that the FedGA-Meta system satisfies the stated properties (Guendouzi et al., 2025).

Lastly, S40 first utilizes active automata learning using Angluin’s L* algorithm to learn a minimal Deterministic Finite Automata (DFA) abstracting an RNN’s behavior (Khmelnitsky et al., 2021; Angluin, 1987). Statistical Model Checking is then used to determine whether the DFA abstraction violates a specification. When violations are found, the method checks whether the RNN truly violates the property or whether the DFA abstraction inaccurately represents the RNN, refining the learned DFA accordingly (Khmelnitsky et al., 2021).

### 4.5 Runtime verification approaches

Broadly, Runtime Verification is a lightweight formal method applied at runtime to provide rigorous guarantees of property adherence for a system. Runtime Verification provides precise information on the runtime behavior of the monitored system at the cost of limited execution coverage. Within this method, safety properties are specified in a formal language, a monitor is generated from the formal specification, and the monitor is instrumented to extract information from the system to verify property adherence against the system trace (Bartocci et al., 2018).

S8 combines runtime assurance with Theorem Proving and SMT-solving to formally verify an airborne collision avoidance system that utilizes an NN for decision making (Cofer et al., 2022). The runtime monitor evaluates the flight plan generated by the collision avoidance system and contains a safe backup planner. The results of the evaluation are then fed into a decision logic component which selects a backup flight plan that ensures safe flight. The decision component decides on a safe flight plan based on a tabular specification of safety rules. The decision logic code is synthesized from a formal specification, and formal proofs of correctness are produced after each step during synthesis. Additionally, the runtime monitor is modeled using the Architecture Analysis and Design Language (AADL) and the study formally analyzes the monitor, using SMT-solving, against safety properties. The Assume Guarantee Reasoning Environment (AGREE) is used to analyze the runtime assurance architecture (Cofer et al., 2022).

Similarly, S16 utilizes both Theorem Proving and runtime monitoring within a modified formally constrained RL framework (Hunt et al., 2021). The study introduces Verifiably Safe Reinforcement Learning (VSRL). Rather than assuming a perfect simulator state, VSRL first trains a lightweight object detector, using only a handful of labeled examples, to extract the positions of safety-critical objects from raw Red Green Blue frames. Instead of embedding constraints directly into the RL algorithm, VSRL wraps the original environment with a safety guard. Whenever the agent proposes an unsafe action, that action is replaced at runtime by a uniformly sampled safe one. The authors show that this method produces a refined MDP in which all actions are safe and whose optimal policies exactly correspond to the best safe policies in the original problem. The authors also use dL to model the environment and controller as hybrid programs. The safety properties are then specified and proven in the dL proof calculus, thus providing a formal certificate of safety from the starting safe state (Hunt et al., 2021).

S38 also focuses on RL-based systems and formally verifies an RL agent implemented via MATLAB Simulink’s RL toolbox (Adelt et al., 2021). The RL component’s acceptable behavior is specified as a hybrid contract in dL, constraining which actions are permitted in which observed states. The Simulink model is transformed into a dL hybrid program where the RL agent is represented as a monitored nondeterministic choice over safe actions consistent with the contract. Safety properties are proven deductively for the entire closed-loop system, and runtime monitors ensure the trained agent’s executed actions satisfy the contract during simulation and training.

S10 performs runtime monitoring of multiple parallel ML models for Over-The-Air (OTA) updates in CPSs (Guissouma et al., 2023). An initial ML model, such as an Artificial Neural Network (ANN), is first deployed after training and the first validation phase. While operating, new data may be added to the original training base, requiring an updated ML model to be sent OTA to the vehicle. As a result, a new version of the ML model is retrained. Further, multiple models may be retrained with different configurations under the new training data. These newly trained versions are then deployed to the vehicle as Shadow Versions (SVs), where each model runs in parallel to the Active Version (AV). While all versions are running, online monitoring to ensure safety is simultaneously performed on all models, and safety properties are expressed in STL. The data during monitoring is collected and analyzed, and when one SV is found to be more robust or safe than the AV, the AV is replaced with that SV. The AV serves as the baseline for future updates (Guissouma et al., 2023).

On the other hand, S11 incorporates formal symbolic reasoning into the construction of runtime monitors for DNNs (Cheng, 2021). The monitors are based on building an abstraction out of neuron activation patterns from the training data. In order to accomplish this, a set of neurons to be monitored is selected. Then, feature vectors are formed by taking the values of all monitored neurons for each input into the DNN. The monitor is constructed algorithmically by building a compact set representation that contains all feature vectors. The result is a sound guarantee, in that a warning over an input means that there is no close input within the training dataset. However, one issue with this approach is that in real-world deployment, the method can yield many false alarms. As a result, the study proposes a different monitor construction algorithm with robustness guarantees (Cheng, 2021).

On a separate note, S13 applies Runtime Verification to verify ML-driven chatbots (Ferrando et al., 2023). A runtime monitor is placed outside of a chatbot environment to ensure that the interaction between the user and chatbot follows a specified interaction protocol. The study presents Runtime Verification for Rasa (RV4Rasa), a framework for the runtime verification of chatbots developed in Rasa. These chatbots can be run locally on a computer rather than solely on the cloud. However, the framework can be extended to other chatbot environments. RV4Rasa checks, based on structured data generated during the Natural Language Understanding (NLU) step, whether the user and chatbot follow a specified interaction protocol (Ferrando et al., 2023).

Lastly, S18 develops runtime monitors analyzing the input and output channels of black-box neural networks, especially CNNs and ANNs (Tripuramallu et al., 2024). Safety properties are formalized as Valued Discrete Timed Automata (VDTA), and the runtime monitors are synthesized from the VDTAs. The runtime monitor operates over the inputs into the CNN and the outputs of the CNN.

### 4.6 Shielding techniques

A shield is a reactive system implemented alongside the learning agent that enforces safety properties specified in a formal language. The learning agent observes the environment and selects an appropriate action, which is then checked by the shield and corrected if the chosen action is deemed unsafe (Newcomb et al., 2024). Note that shielding is also utilized in S15 and S44, however, shielding was not the primary formal technique presented in these studies and they are therefore both discussed in Section 4.4.

S17 presents a dynamic shielding mechanism for MARL where shields dynamically split and merge depending on agent behavior, promoting collaboration between agents to ensure safety (Xiao et al., 2023). When agents are at risk of conservative behavior, such as when their shields block them from taking action due to lack of coordination, the independent shields can merge into one shield that maintains the safe behavior of the agents. When the agents move apart from each other, the shields split apart into multiple shields.

The authors present an algorithm for shield synthesis called K-Step Look Ahead Shields, which is a variant of traditional shield synthesis. The result is potentially improved computational efficiency when shields dynamically centralize and decentralize, as well as improved coordination among agents. The authors use Linear Temporal Logic to formalize safety specifications. They also theoretically prove that their shield synthesis technique guarantees safety (Xiao et al., 2023).

### 4.7 Control barrier function methods

Control Barrier Function (CBF) methods assign a scalar safety score to each state. The safe set is then the super-level set containing all states with scores above a safety threshold, e.g., zero. A function is a CBF if, for every safe state, there exists at least one action that guarantees the next state remains in the safe set. Controllers can then use this condition as a safety filter to choose actions that keep trajectories inside the safe set. S7 reduces a learned Value Function (VF) used within RL into a CBF and verifies that the CBF is valid. If the CBF is valid, the CBF may be used as a formal safety certificate for the RL policy. Experiments validate that learned CBFs as safety filters or certificates are feasible through RL (Tan et al., 2024).

### 4.8 Risk verification methods

Risk verification is a data-driven formal verification approach that, from system execution traces, computes high-confidence bounds on statistical risk metrics over the distribution of robustness values for a given formal specification. The method therefore quantifies the tail risk—the risk from the extreme end of the robustness distribution—and the severity of potential violations (Cleaveland et al., 2022).

S24 applies risk estimation to NN controllers (Cleaveland et al., 2022). Safety requirements are formalized as either simple state constraints or STL formulas. The study estimates, from trajectory data, the risk that an NN-controlled stochastic system will violate a formal specification. Then, the study characterizes how that risk changes when the system is perturbed (e.g., due to environmental changes or modeling errors), by deriving bounds based on measures of system closeness. The steps of the verification framework are as follows:

1. Define a robustness score for each run. For any simulated trajectory of the system under the NN controller, compute the distance from violating a safety rule at every time step. Next, take the minimum of those distances over time as the robustness of that run.
2. Treat robustness as a random variable. Because the system is stochastic, a distribution of robustness values is obtained by running many simulations.
3. Choose a risk metric. Rather than caring only about average performance, pick a measure such as Value-at-Risk (VaR) or Conditional VaR to focus on the worst-case tail of the distribution. Estimate an upper bound on that risk metric from the finite sample, with high confidence, using concentration inequalities.
4. Bound the risk under model mismatch. Real systems or richer simulators may differ from the model. The authors show how to upper-bound the increase in risk when moving from the model to the real system by quantifying how much two trajectories can diverge. This step yields a risk-verification gap, which is an explicit amount that must be added to the model’s nominal risk bound to stay safe on the real system (Cleaveland, Tambon et al., 2022).

### 4.9 Comparison with traditional verification approaches

This section answers the second half of RQ1 by detailing how the described formal methods improve upon traditional verification approaches. S1-2, S4, S8-10, S12, S14, S16, S23, S26, S28, S31-32, S36-37, S41 and S43 briefly discuss limitations to traditional verification that warrants the investigation of formal methods for providing correctness guarantees on ML systems. The evidence from these studies supports deeper studies into formal methods as opposed to traditional methods for enforcing safe ML.

S1 briefly discusses how traditional verification techniques on RL-based systems only provide statistical guarantees at most, and more rigorous techniques, such as formal methods, are therefore necessary to ensure the trustworthiness of RL-based systems (Tao et al., 2025). Also related to RL-based systems, S16 mentions that providing a purely statistical guarantee that an RL agent remains within safe states requires an infeasible amount of training data (Hunt et al., 2021).

Regarding NNs and DNNs, S23 also discusses statistical testing and explains how statistical testing of autonomous systems utilizing NNs is insufficient when verifying the safety of these systems (Meyer, 2023). S2 explains that testing an NN on a complete input set is impossible as the input set is often infinite. Therefore, testing is insufficient when verifying an NN (Naseer et al., 2020). Similarly, S4 mentions that traditional verification techniques on DNNs, such as simulations or random noise analysis, are insufficient for providing formal guarantees against errors. Therefore, traditional methods cannot capture the complete set of potential faults, which is necessary for safety critical applications (Samadi et al., 2024). S8 explains that because the behavior of NNs is largely attributed to its training data, it is generally impossible to determine the correctness of a NN through white-box methods or design analysis (Cofer et al., 2022). S9 describes that traditional verification, such as simulation testing, is non-exhaustive by nature. Therefore, incorporating formal methods into the verification process will help obtain additional completeness guarantees of autonomous software (Nenchev, 2025).

On a similar note, S10 mentions that simulation-based verification methods are insufficient for verifying real-time systems, and as a result, some software errors only become apparent after deployment (Guissouma et al., 2023). S14, S28, and S41 explain that traditional verification techniques, i.e., testing, for DNNs are useful for finding input samples that lead to incorrect behavior. However, these techniques cannot prove the absence of input samples leading to incorrect behavior (Zhang et al., 2022; Hafaiedh et al., 2025; Zhang et al., 2023). S26 discusses how human inspection is not a viable verification technique to use for NNs as it is for traditional programming. The complex function representation of a NN makes it challenging to verify through mechanical analysis of important properties (Gros et al., 2022).

Lastly, S12 argues that existing methods for verifying image-based NNs are limited by the high dimensionality and complexity of image inputs. Therefore, these methods face scalability challenges and computational inefficiencies. Additionally, traditional verification techniques often treat NNs as black-boxes, which makes it challenging to understand the decision making within each controller (Parameshwaran and Wang, 2025).

## 5 Analysis and discussion

This section answers RQ2 and RQ3 in respective subsections.

### 5.1 Research question 2

RQ2 asks, “in which domains or applications have these formal methods been successfully employed for safe ML?” Table 4 presents the application areas or domains identified, the number of articles that correspond to that application/domain, and the source IDs. The largest number of articles discuss the formal verification of NN and DNN controllers, followed by RL policies and NNs/DNNs for perception.

**Table 4:** Applications and domains of selected articles.

Application/domain	Number of articles	Sources
RL for 6G networks	1	S1
NN on noisy inputs	2	S2, S29
Black box system verification	1	S3
DNN resilience	1	S4
QNNs	4	S5, S14, S20, S41
DNN controllers	5	S6, S9, S27, S28, S32
RL policies	5	S7, S16, S38, S39, S44
NN controllers	6	S8, S10, S23, S24, S26, S33
NNs/DNNs for perception	5	S11, S12, S31, S34, S35
ML-Driven Chatbots	1	S13
MARL	2	S15, S17
CNNs	2	S18, S37
SDNNs	1	S19
Federated-learning enabled systems	1	S21
LSTMs/RNNs	4	S22, S25, S40, S46
Semantic segmentation NNs	2	S30, S43
NNs/DNNs for regression	2	S36, S42
Spiking NNs	1	S45

### 5.2 Research question 3

RQ3 asks, “What gaps, limitations, and challenges exist within current work combining formal methods and ML safety, and what future research directions can be identified from these gaps?”

#### 5.2.1 Gaps, limitations, and challenges

Scalability and computational efficiency remain significant issues, even though many of the articles consider these issues in their designs. Specifically, the application of formal methods to complex and large NNs is a particular gap in the literature, as the articles focus on small or medium sized networks. Additionally, time complexity is often left unquantified, and there is a need to perform formal analysis on the time complexity of the solutions. Further, many evaluations are confined to small, domain-specific benchmarks and have not been validated in real-world scenarios, limiting the generalizability from the experiments to the real world.

Especially related to Model Checking, the abstraction of NN/DNN behavior may lead to missed behavior from the real system, resulting in potential gaps during evaluation. Therefore, accurate abstraction methods that retain DNN behavior is a notable challenge. Nearly all approaches apply formal methods after model training, with the exceptions of S15 and S16, with little integration back into the training loop. S1 suggests using Model Checking results to refine the RL model as future work (Tao et al., 2025).

On a separate note, S13, focusing on formally verifying ML-driven chatbots, assumes that the NLU step produces structured information about the user’s request after a query, which may not apply to all chatbots (Ferrando et al., 2023). There is therefore a gap in formally verifying Large Language Models (LLMs) or other ML-driven chatbots that lack explainability. Lastly, the complexity of combining advanced ML methods with formal techniques demands steep technical expertise, potentially hindering adoption. Along this same note, there is little discussion of integration with mainstream ML frameworks or deployment pipelines, which may delay uptake by practitioners beyond specialized research settings.

#### 5.2.2 Future research directions

Each article provides possible future work to pursue for its respective solution, and readers are encouraged to explore these for specific future directions on frameworks of interest. This section instead focuses on high level future research directions that can be gleaned from the overall body of literature.

One area of future work is developing integrated training-verification loops. Specifically, extending beyond post-hoc verification to embed formal checks during model training, which allows for corrective adjustments in real time. Additionally, investigating scalability is a significant area of future work. The formal techniques discussed in this SLR can be extended to larger, more complex networks, real-world networks, and diverse datasets to assess practical viability. The performance of the various formal verification frameworks can also be compared when applied to large-scale networks to further identify scalability gaps in the literature.

Algorithms can be developed to automatically select parameters that balance precision versus performance. Several of the current solutions require human intervention to select parameters during the formal verification process, such as S3, which requires expertise and is error-prone (Nuhu et al., 2022). Further, standardized metrics and benchmarks are an area of future work. Establishing common datasets, network architectures, and evaluation metrics would enable fair comparison across tools.

Hybrid methods, possibly combining multiple techniques or adding an additional formal refinement loop, are a potentially significant area of future exploration. Complementary techniques may be combined to leverage the unique benefits of each technique. For example, Statistical Model Checking can be combined with existing formal approaches to tighten confidence in safety guarantees. Alternately, solutions that blend off-line formal guarantees with on-line data-driven monitors or shields are an interesting area of future work.

Researchers can explore cloud-based architectures to offload heavy verification tasks, support large-scale monitoring, and reduce on-board overhead. Additionally, the formal methods and ML communities would benefit from well documented, modular toolkits and reference implementations to lower the barrier for adoption and facilitate reproducible experiments. Ideally, these toolkits can plug into common ML workflows, e.g., TensorFlow or PyTorch, with minimal instrumentation (TensorFlow, 2025; PyTorch, 2025).

Lastly, with the explosion of LLMs, exploring additional techniques for the formal verification of ML-driven chatbots is of interest. This is especially relevant for techniques that either aid in explainability or are effective without relying on structured information from the NLU step.

## 6 Quality assessment

**Figure 7:**
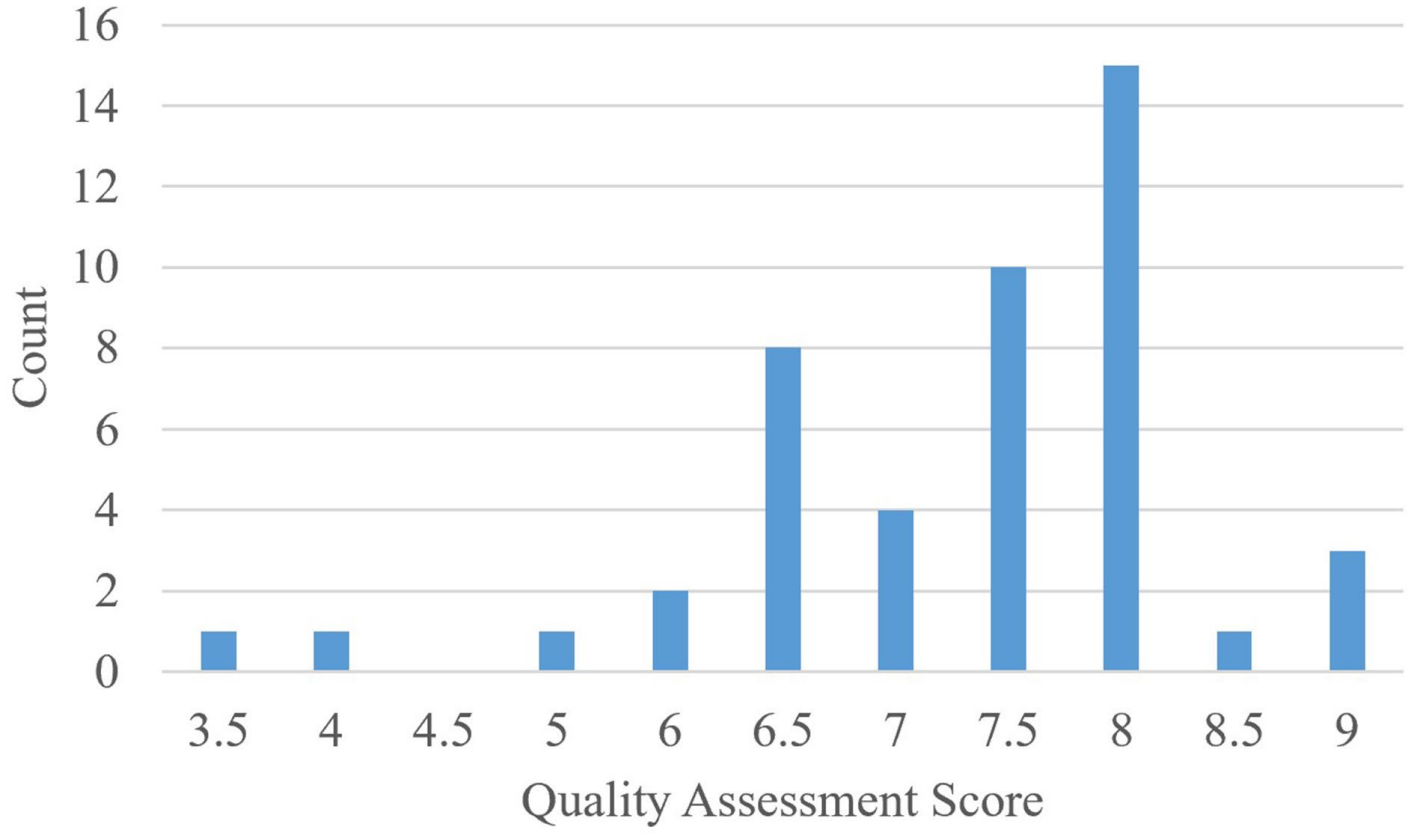
Quality assessment score distribution of the selected articles.

A total score for each paper was obtained by summing the scores of either 0, 0.5, or 1 for each field of the quality assessment checklist. The total score is therefore out of nine points as there are nine total fields. Figure 7 presents the distribution of total scores among the selected 46 articles. Notably, of the 46 articles, only seven explicitly discussed limitations, challenges, and threats to validity (field 6). Six articles partially discussed limitations.

## 7 Related work

Several related surveys and literature reviews have been conducted, and this section discusses the most recent (within the last five years) related work. Krichen et al. survey formal methods approaches for the validation and verification of ML systems (Krichen et al., 2022). When discussing validation, the survey focuses on literature utilizing formal methods for validating data preparation and training phases. On the verification of ML systems, the formal methods for NNs, Decision Tree Ensembles, and Support Vector Machines are surveyed (Krichen et al., 2022). Similarly, Larsen et al. (2022) focus both on applying formal methods to ML systems to aid in their adoption within safety-critical domains and applying ML to formal methods to increase the scalability of formal methods to larger problems. Regarding the former, the authors survey formal methods for improving the explainability of ML systems and the verification of ML systems (Larsen et al., 2022).

Tambon et al. (2022) along with Meyer and Oosthuizen (2023), both contribute SLRs in topics tangential to this work. Tambon et al. (2022) discuss the challenges related to the certification of ML-based safety-critical systems and conduct an SLR on literature between 2015 and 2020. The authors identify the following as the main pillars of ML certification: Robustness, Uncertainty, Explainability, Verification, Safe Reinforcement Learning, and Direct Certification. Comprehensive analyses are provided for each of these areas. When discussing verification, the authors focus on formal methods and empirically guided testing. Similarly to this work, the authors recognize the need to bridge academic research with real-world adoption (Tambon et al., 2022). On the other hand, Meyer and Oosthuizen provide an SLR on the verification and validation of AI-enabled CPS. The review classifies traditional verification methods into multiple categories and mentions that the self-adaptive learning nature of AI requires new verification approaches. The authors briefly discuss formal verification approaches (Meyer and Oosthuizen, 2023).

Separately, Meng et al. specifically survey techniques to improve the adversarial robustness of DNNs from a formal verification perspective (Meng et al., 2022). Adversarial robustness refers to the reliability of ML models to malicious input perturbations. The survey details formal techniques such as SMT-solving, Linear Programming/MILP, interval arithmetic, Reachability Analysis, and more.

Although previous work either systematically reviews or surveys formal verification applied to ML-enabled systems, current literature on the subject is relatively dated, with most of these studies published in Tambon et al., 2022 and the most recent study published in 2023. None of these studies review literature produced after Tambon et al., 2022. Additionally, only two of the studies conducted systematic reviews, and even then, the SLRs are on tangential topics in which formal verification is not the primary focus. There is therefore a gap in the literature for SLRs reviewing state-of-the-art empirical research in safe ML through formal verification, which this work fills.

## 8 Strengths and limitations

This study contributes a current and detailed review on literature in formal methods for safety-critical ML, including thorough discussions on gaps, limitations, challenges, and future work. Additionally, the authors conduct a thorough quality assessment of all articles and present the quality assessment score distribution to readers for reference. Limitations to this study include that the SLR was focused specifically on four digital library sources. Work contributed to other digital libraries was therefore not reviewed. Similarly, non-peer reviewed work, such as those published on ArXiv or ResearchGate, were not reviewed. Additionally, including testing and traditional verification in the PICOC terms limited the scope of the review and excluded articles that do not explicitly include these terms.

## 9 Conclusion

This SLR presents a comprehensive and detailed overview of the state-of-the-art in safe ML through the application of formal methods. By surveying 46 peer-reviewed studies across eight distinct categories, this work synthesizes both the theory and practical implementations that have emerged between 2020 and mid-2025. As a result, this work highlights both the diversity of formal verification techniques currently in use and the increasing scholarly interest and maturity of this research area. Other similar reviews do not capture work produced after 2022 and contribute mostly unsystematic surveys.

The analysis of gaps, limitations, and challenges reveals several areas for investigation that can help guide future research. By outlining opportunity areas for future work, this review serves not only as a record of existing contributions but also as a roadmap for researchers seeking to advance the safety of ML systems. Future researchers may use this work to both familiarize themselves with the current state of the research and determine where in the field to contribute new work.

## Data Availability

The original contributions presented in the study are included in the article/Supplementary material, further inquiries can be directed to the corresponding author.
