# Supplementary material for: Formal methods for safety-critical machine learning: a systematic literature review
Source: Front Artif Intell. 2026 Feb 18;9:1749956. doi: 10.3389/frai.2026.1749956 (PMC12956799; doi:10.3389/frai.2026.1749956)
Supplement: Supplementary file 1 [file Table_1.docx]

Supplementary Material

# Supplementary Tables

Supplementary Table 1 presents the data extraction form. Supplementary Table 1 also displays an example of data extraction using values and notes from Nenchev, a study chosen for data extraction and analysis (Nenchev, 2025). Note that the amount of data extracted from articles varied greatly, and Nenchev was chosen as the example because the extracted data for this article was the most concise.

**Supplementary Table 1.** Data Extraction Form

| **Data Item** | **Value** |
| --- | --- |
|  | *Bibliographic Information* |
| Study identifier | S9 |
| Authors (first six only) | Vladislav Nenchev |
| Database Source | IEEE Xplore |
| Literature type | Conference |
| Name of conference | International Symposium on System Integration (SII) |
| Name of journal | N/A |
| Name of workshop | N/A |
| Country of origin | Germany |
| Year | 2025 |
| Citation count | 1 |
|  | *Technical Information* |
| Application/domain | Formally verifying automated driving behavior represented by a Deep Neural Network (DNN). |
| Research Questions or Aim, Goals, Problem, Objectives, Motivation, Purpose | Porting software onto a vehicle is an error-prone task that may benefit from formal verification to ensure Automated Driving (AD) software remains safe when deployed on varying vehicle hardware. Sometimes, over-the-air software updates are necessary to update vehicle software after a customer has purchased the vehicle, which results in considerable verification challenges due to the complexity of vehicle hardware and software and their interactions. Formal methods, especially applied to automatic portability checking, is an important avenue for exploration to apply rigorous checks on ported AD software on various Vehicle Hardware Configurations (VHCs). |
| Formal method(s) used | Bounded Model Checking |
| AI/ML model used | DNNs |
| Application of formal method to AI/ML model | Bounded Model Checking of a simplified DNN. |
| Description of solution design | Bounded Model Checking is applied to a simplified version of a DNN modeling Adaptive Cruise Control behavior. This is not the primary focus of the paper and is briefly discussed. |
| Description of experimental design | Two controllers are tested: one traditional controller based on Model Predictive Control and one DNN-based controller. The Deep Deterministic Policy Gradient algorithm is used to learn the DNN. |
| Summary of experimental results (quantitative) | The Neural Network Controller was only verified successfully (no counterexample found) for one out of the three vehicle configurations. The other two configurations resulted in counterexamples. VHC 1 completed in 16.4 minutes. VHC 2 completed in 25.4 minutes. VHC 3 completed in 30.1 minutes. |
| Summary of experimental results (qualitative) | N/A |
| Key findings and analysis | The approach is overall validated by the experiments, as the portability of the controllers was either successfully confirmed or counterexamples were produced. Results show that automated checking also occurs in only minutes. |
| Comparison to traditional verification techniques | The author mentions that traditional verification, such as simulation testing, is non-exhaustive by nature. Therefore, incorporating formal methods into the verification process will help obtain additional completeness guarantees of the AD software. |
| Limitations, challenges, threats to validity | The model checked within the framework is a simplified model and does not describe all behaviors of the actual system. Therefore, there may be gaps in the evaluation. The proposed approach should therefore be used alongside other verification techniques and cannot be relied upon alone. |
| Identified future work | - Include statistical verification methods alongside the proposed approach to improve the verification rigor.  - Consider low level code, such as drivers and OS routines, in the verification procedure.  - Model additional sensor effects and explore incorporating other automotive controllers. |
| Additional Notes | Note that the author does not directly apply formal verification directly on the DNN. Instead, the study tests a number of finite states within the Operational Design Domain, model check a simplified version of the DNN and finally use a dedicated DNN verifier to verify that the DNN produces a safe action for each state in the safe set. |

# References

Nenchev, V., 2025. *One Stack, Diverse Vehicles: Checking Safe Portability of Automated Driving Software.* Munich, Germany, s.n.
