# Supplementary material for: Formal methods for safety-critical machine learning: a systematic literature review
Source: Front Artif Intell. 2026 Feb 18;9:1749956. doi: 10.3389/frai.2026.1749956 (PMC12956799; doi:10.3389/frai.2026.1749956)
Supplement: Supplementary file 2 [file Table_2.docx]

Supplementary Material

# Supplementary Tables

Supplementary Table 2 presents a summary of each selected article within the Systematic Literature Review. The “S” in the identifier stands for “Study”.

**Supplementary Table 2.** Summary of Selected Articles

| **ID** | **Authors** | **Formal Method(s)** | **ML Method** |
| --- | --- | --- | --- |
| S1 | Tao et al. (Tao, et al., 2025) | Probabilistic Model Checking | Reinforcement Learning (RL) |
| S2 | Naseer et al. (Naseer, et al., 2020) | Symbolic Model Checking | Neural Networks (NNs) |
| S3 | Nuhu et al. (Nuhu, et al., 2022) | Satisfiability Modulo Theory (SMT)-based Approach | DNNs |
| S4 | Samadi et al. (Samadi, et al., 2024) | SMT-based Approach | DNNs |
| S5 | Mistry et al. (Mistry, et al., 2022) | MILP | Quantized Neural Networks (QNNs) |
| S6 | Zhao et al. (Zhao, et al., 2023) | Polynomial Inclusion Computation and Barrier Certificate Generation | DNNs |
| S7 | Tan et al. (Tan, et al., 2024) | Control Barrier Functions | RL |
| S8 | Cofer et al. (Cofer et al., 2022) | Runtime Verification, Theorem Proving, SMT-Solving | NNs |
| S9 | Nenchev (Nenchev, 2025) | Bounded Model Checking | DNNs |
| S10 | Guissouma et al. (Guissouma, et al., 2023) | Runtime Verification | NNs |
| S11 | Cheng (Cheng, 2021) | Runtime Verification and Symbolic Reasoning | DNNs |
| S12 | Parameshwaran and Wang (Parameshwaran & Wang, 2025) | Reachability via Bound Propagation | Image-Based NNs |
| S13 | Ferrando et al. (Ferrando, et al., 2023) | Runtime Verification | ML-Driven Chatbots |
| S14 | Zhang et al. (Zhang, et al., 2022) | ILP | QNNs |
| S15 | El Mqirmi et al. (El Mqirmi, et al., 2021) | Model Checking, Shielding (Reactive Synthesis) | Multi-Agent Reinforcement Learning (MARL) |
| S16 | Hunt et al. (Hunt, et al., 2021) | Theorem Proving, Runtime Verification | RL |
| S17 | Xiao et al. (Xiao, et al., 2023) | Shielding (Reactive Synthesis) | MARL |
| S18 | Tripuramallu et al. (Tripuramallu, et al., 2024) | Runtime Verification | Convolutional Neural Networks (CNNs) |
| S19 | Das et al. (Das, et al., 2025) | SMT-based Approach | Sigma-Delta Neural Networks (SDNNs) |
| S20 | Bachiri et al. (Bachiri, et al., 2025) | SMT-based Approach | QNNs |
| S21 | Guendouzi et al. (Guendouzi, et al., 2025) | Model Checking | Federated Learning-Based Industrial Cyber Physical Systems (CPSs) |
| S22 | Choi et al. (Choi, et al., 2025) | Star-Set Reachability | Recurrent Neural Networks (RNNs) |
| S23 | Meyer (Meyer, 2023) | Mixed-Monotonicity Reachability Analysis | NNs |
| S24 | Cleaveland et al. (Cleaveland, et al., 2022) | Risk Verification using Constraints and Signal Temporal Logic (STL) | NNs |
| S25 | Liang et al. (Liang, et al., 2024) | Model Checking | RNNs |
| S26 | Gros et al. (Gros, et al., 2022) | Statistical Model Checking | NNs |
| S27 | Elboher et al. (Elboher, et al., 2024) | Abstraction/Refinement for SMT-Solving | DNNs |
| S28 | Hafaiedh et al. (Hafaiedh, et al., 2025) | Reachability Analysis | DNNs |
| S29 | Zhong et al. (Zhong, et al., 2023) | Over-Approximation using convex hull | NNs |
| S30 | Pal, Lee, and Johnson (Pal, et al., 2023) | Set-based Reachability Analysis | Semantic Segmentation NNs |
| S31 | Tang et al. (Tang, et al., 2023) | Over-Approximation using convex hull | NNs |
| S32 | Zhao et al. (Zhao, et al., 2022) | SMT-based Approach | DNNs |
| S33 | Zhou and Tripakis (Zhou & Tripakis, 2024) | SMT-based Approach | NNs |
| S34 | Ashok et al. (Ashok, et al., 2020) | Abstraction and Over-Approximation | NNs |
| S35 | Paterson et al. (Paterson, et al., 2021) | SMT-based Approach | DNNs |
| S36 | Vidot et al. (Vidot, et al., 2022) | MILP | NNs |
| S37 | Kirov et al. (Kirov, et al., 2023) | Reachability Analysis and Abstract Interpretation | CNNs |
| S38 | Adelt et al. (Adelt, et al., 2021) | Runtime Verification | RL |
| S39 | Genin et al. (Genin, et al., 2021) | SMT-based Approach | RL |
| S40 | Khmelnitsky et al. (Khmelnitsky, et al., 2021) | Statistical Model Checking | RNNs |
| S41 | Zhang et al. (Zhang, et al., 2023) | Reachability Analysis and MILP | DNNs |
| S42 | Pal, Lopez, and Johnson (Pal, et al., 2023) | Star-Set Reachability Analysis | DNNs |
| S43 | Tran et al. (Tran, et al., 2021) | ImageStar Reachability Analysis | Semantic Segmentation NNs |
| S44 | Adelt et al. (Adelt, et al., 2023) | Statistical Model Checking | RL |
| S45 | Banerjee et al. (Banerjee, et al., 2023) | SMT-based Approach | Spiking NNs |
| S46 | Moradkhani et al. (Moradkhani, et al., 2023) | SMT-based Approach | LSTMs |

# References

Adelt, J. et al., 2023. *Shielded Learning for Resilience and Performance Based on Statistical Model Checking in Simulink.* Cham, Switzerland, Springer, Cham.

Adelt, J., Liebrenz, T. & Herber, P., 2021. *Formal Verification of Intelligent Hybrid Systems that are Modeled with Simulink and the Reinforcement Learning Toolbox.* Cham, Switzerland, Springer, Cham.

Ashok, P., Hashemi, V., Křetínský, J. & Mohr, S., 2020. *DeepAbstract: Neural Network Abstraction for Accelerating Verification.* Cham, Switzerland, Springer, Cham.

Bachiri, W., Seladji, Y. & Garoche, P.-L., 2025. Formal Specification and SMT Verification of Quantized Neural Network for Autonomous Vehicles. *Science of Computer Programming,* Volume 245.

Banerjee, S., Ghosh, S., Banerjee, A. & Mohalik, S. K., 2023. *SMT-Based Modeling and Verification of Spiking Neural Networks: A Case Study.* Cham, Switzerland, Springer, Cham.

Cheng, C.-H., 2021. *Provably-Robust Runtime Monitoring of Neuron Activation Patterns.* Grenoble, France, s.n.

Cheng, C.-H., 2021. *Provably-Robust Runtime Monitoring of Neuron Activation Patterns.* Grenoble, France, Design, Automation & Test in Europe Conference & Exhibition (DATE).

Choi, S. W. et al., 2025. Reachability Analysis of Recurrent Neural Networks. *Nonlinear Analysis: Hybrid Systems,* Volume 56.

Cleaveland, M., Lindemann, L., Ivanov, R. & Pappas, G. J., 2022. Risk Verification of Stochastic Systems with Neural Network Controllers. *Artificial Intelligence,* Volume 313.

Cofer et al., D., 2022. *Flight Test of a Collision Avoidance Neural Network with Run-Time Assurance.* Portsmouth, VA, USA, s.n.

Cofer et al., D., 2022. *Flight Test of a Collision Avoidance Neural Network with Run-Time Assurance.* Portsmouth, VA, USA, 2022 IEEE/AIAA 41st Digital Avionics Systems Conference (DASC).

Das, S., Banerjee, A. & Mohalik, S. K., 2025. *Modeling and Verification of Sigma Delta Neural Networks using Satisfiability Modulo Theory.* Seoul, Republic of Korea, s.n.

Das, S., Banerjee, A. & Mohalik, S. K., 2025. *Modeling and Verification of Sigma Delta Neural Networks using Satisfiability Modulo Theory.* New York, NY, USA, Association for Computing Machinery.

El Mqirmi, P., Belardinelli, F. & León, B. G., 2021. *An Abstraction-based Method to Check Multi-Agent Deep Reinforcement-Learning Behaviors.* Richland, SC, s.n.

El Mqirmi, P., Belardinelli, F. & León, B. G., 2021. *An Abstraction-based Method to Check Multi-Agent Deep Reinforcement-Learning Behaviors.* Richland, SC, International Foundation for Autonomous Agents and Multiagent Systems.

Elboher, Y. Y., Cohen, E. & Katz, G., 2024. On Applying Residual Reasoning Within Neural Network Verification. *Software and Systems Modeling,* Volume 23, pp. 721-736.

Ferrando, A., Gatti, A. & Mascardi, V., 2023. *RV4Rasa: A Formalism-Agnostic Runtime Verification Framework for Verifying ChatBots in Rasa.* Seattle, WA, USA, s.n.

Ferrando, A., Gatti, A. & Mascardi, V., 2023. *RV4Rasa: A Formalism-Agnostic Runtime Verification Framework for Verifying ChatBots in Rasa.* New York, NY, USA, Association for Computing Machinery.

Genin, D. et al., 2021. *Formal Verification of Neural Network Controllers for Collision-Free Flight.* Cham, Switzerland, Springer, Cham.

Gros, T. P. et al., 2022. Analyzing Neural Network Behavior Through Deep Statistical Model Checking. *International Journal on Software Tools for Technology Transfer,* Volume 25, pp. 407-426.

Guendouzi, B. S., Ouchani, S., Al Assaad, H. & El Zaher, M., 2025. Ensuring the Federation Correctness: Formal Verification of Federated Learning in Industrial Cyber-Physical Systems. *Future Generation Computer Systems,* Volume 166.

Guissouma, H., Zink, M. & Sax, E., 2023. *Continuous Safety Assessment of Updated Supervised Learning Models in Shadow Mode.* L'Aquila, Italy, s.n.

Guissouma, H., Zink, M. & Sax, E., 2023. *Continuous Safety Assessment of Updated Supervised Learning Models in Shadow Mode.* L'Aquila, Italy, 2023 IEEE 20th International Conference on Software Architecture Companion (ICSA-C) .

Hafaiedh, I. B. et al., 2025. *A High Parallelization Method for Automated Formal Verification of Deep Neural Networks.* Djerba, Tunisia, s.n.

Hafaiedh, I. B. et al., 2025. *A High Parallelization Method for Automated Formal Verification of Deep Neural Networks.* Cham, Switzerland, Springer, Cham.

Hunt, N. et al., 2021. *Verifiably Safe Exploration for End-to-End Reinforcement Learning.* Nashville, TN, USA, s.n.

Hunt, N. et al., 2021. *Verifiably Safe Exploration for End-to-End Reinforcement Learning.* New York, NY, USA, Association for Computing Machinery.

Khmelnitsky, I. et al., 2021. *Property-Directed Verification and Robustness Certification of Recurrent Neural Networks.* Cham, Switzerland, Springer, Cham.

Kirov, D., Rollini, S. F., Guglielmo, L. D. & Cofer, D., 2023. *Formal Verification of a Neural Network Based Prognostics System for Aircraft Equipment.* Cham, Switzerland, Springer, Cham.

Liang, Z. et al., 2024. Qualitative and Quantitative Model Checking Against Recurrent Neural Networks. *Journal of Computer Science and Technology,* Volume 39, pp. 1292-1311.

Meyer, P.-J., 2023. *Reachability Analysis of Neural Networks with Uncertain Parameters.* Yokohama, Japan, s.n.

Meyer, P.-J., 2023. Reachability Analysis of Neural Networks with Uncertain Parameters. *IFAC-PapersOnLine,* 56(2).

Mistry, S., Saha, I. & Biswas, S., 2022. An MILP Encoding for Efficient Verification of Quantized Deep Neural Networks. *IEEE Transactions on Computer-Aided Design of Integrated Circuits and Systems ,* 41(11), pp. 4445-4456.

Moradkhani, F., Fibich, C. & Fränzle , M., 2023. *Verification of LSTM Neural Networks with Non-linear Activation Functions.* Cham, Switzerland, Springer, Cham.

Naseer, M. et al., 2020. *FANNet: Formal Analysis of Noise Tolerance, Training Bias and Input Sensitivity in Neural Networks.* Grenoble, France, s.n.

Naseer, M. et al., 2020. *FANNet: Formal Analysis of Noise Tolerance, Training Bias and Input Sensitivity in Neural Networks.* Grenoble, France, 2020 Design, Automation & Test in Europe Conference & Exhibition (DATE).

Nenchev, V., 2025. *One Stack, Diverse Vehicles: Checking Safe Portability of Automated Driving Software.* Munich, Germany, s.n.

Nenchev, V., 2025. *One Stack, Diverse Vehicles: Checking Safe Portability of Automated Driving Software.* Munich, Germany, 2025 IEEE/SICE International Symposium on System Integration (SII).

Nuhu, A.-R.et al., 2022. *Negative Selection Approach to support Formal Verification and Validation of BlackBox Models' Input Constraints.* Singapore, Singapore, s.n.

Nuhu, A.-R.et al., 2022. *Negative Selection Approach to support Formal Verification and Validation of BlackBox Models' Input Constraints.* Singapore, Singapore, 2022 IEEE Symposium Series on Computational Intelligence (SSCI).

Pal, N., Lee, S. & Johnson, T. T., 2023. *Benchmark: Formal Verification of Semantic Segmentation Neural Networks.* Cham, Switzerland, Springer, Cham.

Pal, N., Lopez, D. M. & Johnson, T. T., 2023. *Robustness Verification of Deep Neural Networks Using Star-Based Reachability Analysis with Variable-Length Time Series Input.* Cham, Switzerland, Springer, Cham.

Parameshwaran, A. & Wang, Y., 2025. *Scalable and Interpretable Verification of Image-based Neural Network Controllers for Autonomous Vehicles.* Irvine, CA, USA, s.n.

Parameshwaran, A. & Wang, Y., 2025. *Scalable and Interpretable Verification of Image-based Neural Network Controllers for Autonomous Vehicles.* New York, NY, USA, Association for Computing Machinery.

Paterson, C. et al., 2021. *DeepCert: Verification of Contextually Relevant Robustness for Neural Network Image Classifiers.* Cham, Switzerland, Springer, Cham.

Samadi, A., Harous, A., Mohamed, O. A. & Boukadoum, M., 2024. *Advanced SEU and MBU Vulnerability Assessment of Deep Neural Networks in Air-to-Air Collision Avoidance Systems via SAT-Based Techniques.* Springfield, MA, USA, s.n.

Samadi, A., Harous, A., Mohamed, O. A. & Boukadoum, M., 2024. *Advanced SEU and MBU Vulnerability Assessment of Deep Neural Networks in Air-to-Air Collision Avoidance Systems via SAT-Based Techniques.* Springfield, MA, USA, 2024 IEEE 67th International Midwest Symposium on Circuits and Systems (MWSCAS).

Tan, D. C. et al., 2024. *Safe Value Functions: Learned Critics as Hard Safety Constraints.* Bari, Italy, s.n.

Tan, D. C. et al., 2024. *Safe Value Functions: Learned Critics as Hard Safety Constraints.* Bari, Italy, 20th International Conference on Automation Science and Engineering (CASE).

Tang, X., Zheng, Y. & Liu, J., 2023. *Boosting Multi-neuron Convex Relaxation for Neural Network Verification.* Cham, Switzerland, Springer, Cham.

Tao, X. et al., 2025. *ReLVaaS: Verification-as-a-Service to Analyze Trustworthiness of RL-based Solutions in 6G Networks.* Bengaluru, India, s.n.

Tao, X. et al., 2025. *ReLVaaS: Verification-as-a-Service to Analyze Trustworthiness of RL-based Solutions in 6G Networks.* Bengaluru, India, 2025 17th International Conference on COMmunication Systems and NETworks (COMSNETS).

Tran, H.-D.et al., 2021. *Robustness Verification of Semantic Segmentation Neural Networks Using Relaxed Reachability.* Cham, Switzerland, Springer, Cham.

Tripuramallu, D. et al., 2024. *Runtime Verified Neural Networks for Cyber-Physical Systems.* Vienna, Austria, s.n.

Tripuramallu, D. et al., 2024. *Runtime Verified Neural Networks for Cyber-Physical Systems.* New York, NY, USA, Association for Computing Machinery.

Vidot, G. et al., 2022. *Formal Monotony Analysis of Neural Networks with Mixed Inputs: An Asset for Certification.* Cham, Switzerland, Springer, Cham.

Xiao, W., Lyu, Y. & Dolan, J., 2023. *Model-based Dynamic Shielding for Safe and Efficient Multi-agent Reinforcement Learning.* London, UK, s.n.

Xiao, W., Lyu, Y. & Dolan, J., 2023. *Model-based Dynamic Shielding for Safe and Efficient Multi-agent Reinforcement Learning.* Richland, SC, International Foundation for Autonomous Agents and Multiagent Systems.

Zhang, Y., Song, F. & Sun, J., 2023. *QEBVerif: Quantization Error Bound Verification of Neural Networks.* Cham, Switzerland, Springer, Cham.

Zhang, Y. et al., 2022. *QVIP: An ILP-based Formal Verification Approach for Quantized Neural Networks.* Rochester, MI, USA, s.n.

Zhang, Y. et al., 2022. *QVIP: An ILP-based Formal Verification Approach for Quantized Neural Networks.* New York, NY, USA, Association for Computing Machinery.

Zhao, H. et al., 2023. *Safe DNN-type Controller Synthesis for Nonlinear Systems via Meta Reinforcement Learning.* San Francisco, CA, USA, s.n.

Zhao, H. et al., 2023. *Safe DNN-type Controller Synthesis for Nonlinear Systems via Meta Reinforcement Learning.* San Francisco, CA, USA, 2023 60th ACM/IEEE Design Automation Conference (DAC).

Zhao, Z. et al., 2022. *CLEVEREST: Accelerating CEGAR-based Neural Network Verification via Adversarial Attacks.* Cham, Switzerland, Springer, Cham.

Zhong, Y., Ta, Q.-T. & Khoo, S.-C., 2023. *ARENA: Enhancing Abstract Refinement for Neural Network Verification.* Cham, Switzerland, Springer, Cham.

Zhou, Y. & Tripakis, S., 2024. *Compositional Inductive Invariant Based Verification of Neural Network Controlled Systems.* Cham, Switzerland, Springer, Cham.
